# Life History, Aggregation and Dormancy of the Rubber Plantation Litter Beetle, *Luprops tristis*, from the Rubber Plantations of Moist South Western Ghats

**DOI:** 10.1673/031.008.0101

**Published:** 2008-01-08

**Authors:** Thomas K. Sabu, K.V. Vinod, M.C. Jobi

**Affiliations:** Litter Entomology Research Unit, P.O. & Research Department of Zoology, St. Joseph's College, Devagiri, Calicut, Kerala, India 673008

**Keywords:** *Hevea brasiliensis*, Tenebrionidae, Coleoptera, home invasion, Mupli beetle, oligopause, reproductive diapause, rubber plantation litter, Kerala, South India

## Abstract

Life history, aggregation and dormancy of rubber plantation litter beetle *Luprops tristis* Fabricius, (Tenebrionidae: Coleoptera) is described from rubber plantation belts in the western slopes of Western Ghats from the south Indian state of Kerala. The life cycle lasted 12 months, including the 5 larval instars lasting 1 month, the 3 day pupal stage, and the adult stage that can last 11 months. The adult stage includes an inactive dormancy phase of 9 months in shelters and 1 month each of active pre-dormancy (feeding) and post-dormancy (feeding and reproduction) phases that occur in rubber plantation litter. Reproductive activities are confined to the post-dormancy phase. With the onset of summer rains, huge aggregations of adults invade residential buildings and enter into a state of dormancy for 9 months. Beetle aggregations were in the range of 0.5 million to 4.5 million individuals per residential building. Dormancy in *L. tristis* is best classified as oligopause, which is intermediate between quiescence and diapause. Adults and larvae feed preferentially on wilted rubber tree leaves. Age-specific variation in mortality during dormancy is distinct with higher survivability for adults that have a longer pre-dormancy period. Generations are non-overlapping.

## Introduction

**Figure 1.**
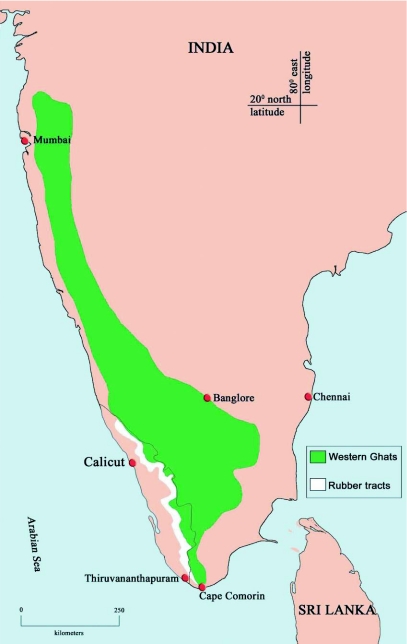
Rubber plantation tracts in the western slopes of south Western Ghats in south India.

*Luprops* beetles (Tenebrionidae: Lagriinae: Lupropini), extend from tropical Africa through Asia and the East Indies to Papua New Guinea and are generally regarded as an inconspicuous litter-dwelling detritivore (Doyen et al. 1990). However, invasion of huge aggregations of the litter dwelling beetle, *Luprops tristis* Fabricius, numbering about 0.5 to over 4 million per residential building following summer showers, and their prolonged stay in a state of dormancy, is a regular event in rubber plantation tracts along the western slopes of the southern region of the Western Ghats (Figure 1). Their high abundance in the range of 200 per m^2^ of litter or about 20 million per hectare in rubber plantations in the vicinity of over wintering shelters (Sabu 2006), illustrates the regional importance of *L. tristis* as a nuisance species. Half a million hectares of rubber plantations are present along the western slopes of the Western Ghats in the south Indian states of Kerala and Tamil Nadu (Agricultural statistics 2005–2006, Hindu 2004). Even if hypothetically only 20% of rubber plantations are mature, with a litter layer thick enough to support beetles, estimates of their abundance reach an astonishing 50 billion. With their litter feeding habits, harmless nature, nocturnal surface activity and diurnal passivity in lower litter layers they would have remained inconspicuous facilitators of litter decomposition and nutrient cycling in monoculture rubber plantation forests in the region. However, their massive seasonal invasion into residential buildings makes them the most dreaded beetles to farming communities surrounding rubber plantations. Massive aggregations numbering approximately 70,000 individuals and dormancy by another tropical beetle (*Stenotarsus rotundus* Arrow, Endomychidae) was earlier reported from the tropical forests in Panama (Wolda and Denlinger 1984; Tanaka et al. 1987; Denlinger 1994). Yet, their sheer abundance along with extensive incidence of such aggregations in the region and the persistent use of a subset of buildings in each locality as shelter for more than two decades makes the present case unique.

The continued presence and attraction of these beetles towards light, following overnight invasion into buildings is a frustrating nuisance for local people. Clusters of several hundred to thousands crawl inside the living rooms and fall off into beds and food from ceilings. Subsequently they congregate in dark, undisturbed areas such as attics and wall voids and remain dormant for several months. They do not sting or bite, but when disturbed, (such as picking them off the walls or when they are squashed or pressed against while sleeping), they release an irritating odoriferous phenolic secretion that causes a burn to the skin. The hapless rubber farming community and the authorities of the residential educational institutions in the rubber belts apply broad-spectrum insecticides to kill the aggregated masses of beetles with full awareness of the risks involved in indoor application of insecticides.

**Figure 2.**
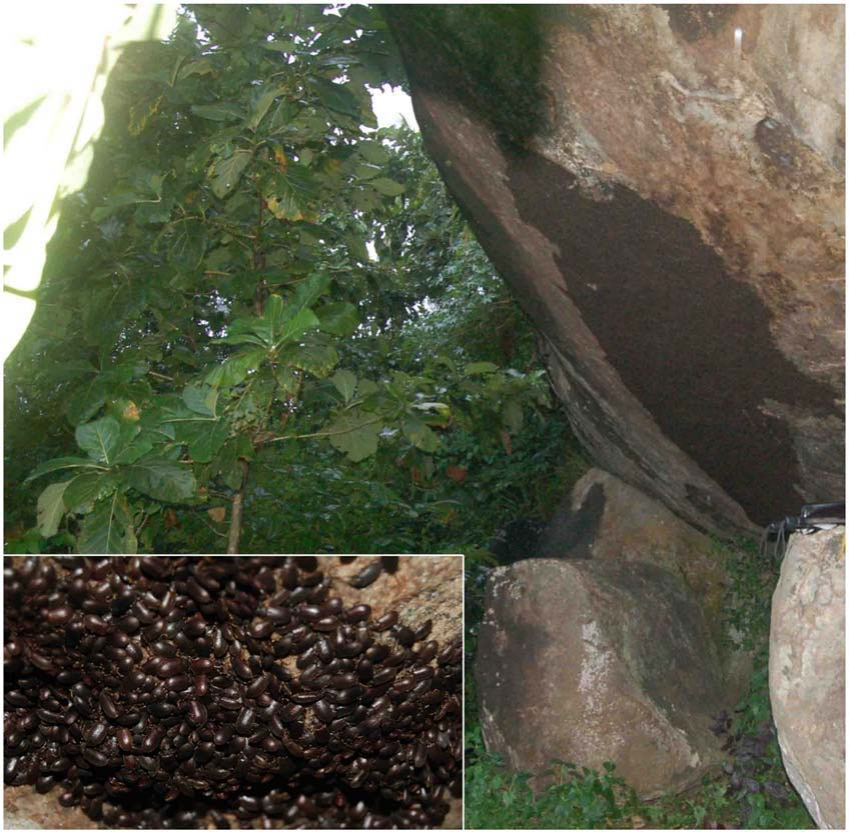
Natural aggregation of *L. tristis* below boulders.

The low-income estate workforce living in the vicinity of plantations in one room coconut palm-thatched, low-height sheds are the worst affected. Small sheds made of stacked coconut fronds are the preferred shelters of these beetles and the beetles are strongly attracted to kerosene lamps used by low-income labourers. In addition to traditional tile roofed wooden buildings and thatched sheds, *L. tristis* aggregates in a variety of other over-wintering quarters in the premises of residential buildings in rubber plantation tracts including hay stacks in cow sheds, hollow wooden blocks, coconut fronds and coconut husks in firewood stocks, and piles of worn out polyethylene rain guards. Aggregations are also common in crevices below boulders in rubber plantations (Figure 2). Its familiar presence below coconut sheaths in thatched sheds and in firewood stocks, and on woodworks supporting earthen tile roofing in traditional buildings has led to the popular names “Ola prani” and “Oadu vandu” (Ola=coconut fronds; Oadu=clay tile; prani & vandu= beetle in the regional language) in north Kerala. In central and south Kerala, they are known as “Mupli vandu”, in reference to the first reporting of such huge aggregations in the rubber estates at Muplium in central Kerala during the 1970s.

Despite three decades of widespread presence in the region, except for short notes on developmental stages and status as a nuisance pest (Narendran 1998; Jose 2003; Thomas and Jacob 2005) no details about the biology, behavior and dormancy of *L. tristis* are available. Its amazingly synchronous massive infestation, abundance and selection of specific buildings as shelter for more than 20 consecutive years, remains as a curious ecological phenomenon to biologists whereas it is a matter causing agony and concern to the affected people in rubber plantation belts. In the present study, comprehensive information on the life cycle, aggregation, behavior and dormancy of the beetle based on studies in both nature and laboratory conditions are provided. Environmental conditions during diapause profoundly influence its duration and therefore it is erroneous to relate laboratory derived data to field situations in studies involving diapause (Tauber and Tauber 1976). Hence, we conducted experiments under laboratory and field conditions and monitored the activities of aggregations settled in natural conditions. These details may assist in the development of efficient strategies for controlling the population build up of *L. tristis* by the efforts of scientific community and rubber planters, and provide additional data on the biology and ecology of this species.

## Materials and Methods

Studies were conducted at Devagiri college campus located 6 km east off the Malabar Coast at Calicut (11° 15′ N, 75° 48′ E), in the Kerala state of India (Figure 1). Calicut receives an average annual rainfall ca 200 cm (CWRDM 2005) and has a relative humidity of 80%. The northeast monsoon from October to November supplements the June to September southwest monsoon in the region. An isolated 4 hectare rubber (*Hevea brasiliensis* (Wild.ex Adr. De Jus) Muell. Arg. of RRII 105 clone) plantation is present on the college campus located midway (50 m amsl) between the coastal area and the rubber plantation belts in the foothills of Wayanad region of Western Ghats. Three essential components for the study were available on the college campus: a rubber plantation with previous history of *Luprops* incidence, a college dormitory building in the vicinity of this plantation with a history of beetle invasion, and a laboratory setup. These enabled simultaneous studies under field and laboratory conditions. Field studies were conducted in an outdoor shelter consisting of a 5 × 5 m plot covered with a few coconut fronds in the middle of the plantation. This shelter was provided because plantations are devoid of leaves in December–January after leaf shedding and direct sunlight reaches the floor of the plantation. Beetles confined in experimental setups in the field do not have the liberty to freely choose an ideal spot under hot dry conditions during the daytime. Experiments in aggregation shelters were done in the dormitory building.

### Life history studies

Life history studies were conducted in experimental setups placed in plantations, residential buildings and the laboratory from December 2004 to June 2007.

#### a) Life history studies in laboratory setups

A preliminary study was carried out under the outdoor shelter and in the laboratory during 2004–05 to determine the feasibility of 1) rearing *L. tristis* in clay vessels and plastic trays filled with field collected soil and litter, 2) maintaining dormant beetles in clay vessels and wooden boxes, 3) inducing dormancy by spraying of water, and 4) identifying the period of arousal from dormancy. These trials revealed that arousal from dormancy and the post-dormancy return of beetles coincide with the onset of annual rubber tree litter fall in early December. Hence, activities of dormant beetles in the dormitory building were monitored until their exodus to rubber plantations ahead of the onset of annual litter fall in rubber plantations in the second week of December. Approximately 300 person hours were spent in plantations and in dormitory buildings recording the behaviour of *L. tristis* during dormancy and return to rubber plantations.

Twenty randomly selected 1 m^2^ quadrants of rubber litter from the plantation were sifted on alternate days with the onset of annual litter fall in December to determine the return of beetles to the field. 100 males and females were randomly sorted from the collections. Sexing was based on the presence or absence of the sternal notch on 8th sternite (Vinod et al. in press). Females were marked with white acrylic paint on elytra.

Sorted males and females were kept in four mesh-topped large clay vessels (Twenty-five males and twenty-five females in each vessel) (13 × 35 cm) half-filled with soil and freshly fallen rubber plantation litter. Clay vessels were transferred to a wooden growth chamber (1.3 × 1.6 × 0.6 m) with fine meshed side windows, front glass panel and provisions for shading. The exterior of clay vessels and litter inside were sprayed with water to simulate the wet litter conditions prevailing in rubber plantations from the settling of dewdrops in December and January.

As soon as mating pairs were observed in the litter floor, paired males and females were transferred to labeled plastic vials (5.6 × 7.2 cm) with a thin layer of charcoal and plaster of Paris mixture at the bottom. Vials were kept together in tall rectangular plastic trays (43 × 32 × 8 cm) and placed in the wooden chamber. Thirty replicate pairs were maintained. For each pair, mating, duration of pre-oviposition, frequency of oviposition and fecundity were recorded.

One hundred eggs laid during a 12-hr period were transferred to 5 petri dishes (9 × 1.5 cm) (20/dish) and monitored at 6-hr intervals. The number of eggs hatched and the duration of egg development were recorded. Pairs of neonate larvae hatched within a 6-hr period were transferred to labeled plastic vials (5.6 × 7.2 cm) with a moist fine hairbrush and provided fresh litter as food source. Two larvae per container were used, to make it possible to follow the development of successive larval instars. This procedure also avoided the inhibitory effect of crowding on larval development in tenebrionids (Tschinkel and Wilson 1971). Development of paired larvae up to adult eclosion was monitored. Groups of 10 vials (i.e. 20 individuals) were treated as a single sample. Five such groups were maintained. Thus, a total of 100 larvae in 50 vials were available for analysis. A parallel stock of 100 individuals was maintained to replace the individuals lost by mortality in different stages. Larvae were monitored at every 12-hr interval, with the development time of instars determined from the exuviae left behind. The duration of larval, pre-pupal and pupal stages was recorded. Larval and pupal survival was measured as the percentage of emerging larvae or pupal stages.

All newly eclosed (teneral) adults that emerged during a 3 day period were pooled into a single emergence class. Those that eclosed during a 31–33 day developmental period were labeled as emergence class I, 34–36 day period as emergence class II, 37–39 day period as class III and 40–45 day period as class IV. Two individuals that eclosed on 44 and 45 day were added to the emergence class IV to limit the number of emergence classes.

Beetles from each emergence class were transferred to a wide mouthed circular clay vessel (35×13 cm) arranged with a layer of top soil, and rubber litter as detailed above, and closed with fine meshed plastic net. Rainfall inside the experimental chambers was simulated by spraying water until the leaves and upper layer of soil inside the clay vessels were as wet as in plantations (about 80% humidity). Humidity inside the vessels was assessed with a thermo-hygrometer (Barigo, www.barigo.de). Spraying was performed synchronously with the onset of summer showers and the mass migration of beetles to the dormitory buildings on April 4th in 2005. A rectangular wooden box (15 × 7 × 3 cm) was placed inside the clay vessel as potential shelter. It was half filled with hay and had a hinged door that was kept half open. Spraying continued until beetles moved into the dry wooden shelter. To prevent clustering of beetles on mesh nets, wet cotton cloth was spread over the mesh net. Clay vessels and the litter inside were kept moist for a week to ensure that aggregated beetles did not return to dry litter in the clay vessels. Subsequently, leaf litter was removed from the vessels, as plantations are devoid of leaf litter until following season. Activities of aggregated dormant beetles in each setup were observed. Mortality was recorded by counting the number of dead beetles at bimonthly intervals. Cages were refilled with thin layer of fresh litter in synchrony with the onset of litter fall in December. After dormancy, beetles were returned to the litter and were individually marked with dots of marker paint on elytra and pronotum (Johnson 2004) using a coding system enabling identification of up to 24 individuals. This enabled estimation of the post dormancy duration of each individual. For each beetle, the date of emergence from dormancy and return to fresh litter was noted. Dead beetles were removed daily and post dormancy duration was recorded. All newly emerged larvae (new generation) inside the setups were removed. After the dormancy period, the beetles were observed until 100% mortality was reached. Egg hatchability, duration and mortality of larval, pupal and adult stages were recorded. Longevity was estimated as the sum of the means of larval, pupal and adult durations.

#### b) Life history studies in natural setups

Twenty eggs of uniform age were collected as described above. Eggs were transferred to a Petri dish in a clay vessel (7 × 30 cm) with fine drainage holes and half-filled with soil and litter. The vessel was buried in the plantation floor with the rim about 1 cm above the soil surface to limit the entry of running rainwater, and covered with muslin cloth supported by iron wires to contain first and second larval instars. Fresh litter was spread over the muslin cloth. We assumed that this design enabled the maintenance of similar insulating conditions for the eggs and larvae placed inside the vessels. Five replicates were thus maintained in the shelter covered with coconut fronds described above.

The exterior of the trays and the litter inside was sprayed with water each morning (7–8 AM). Wetting the litter inside was necessary as the cloth covering prevented entry of dew. The number of eggs hatched was checked at 12-hr intervals. Unhatched eggs were replaced with first instar larvae from the stock culture. When 4th and 5 instar larvae appeared, the cloth topping was replaced with a mesh net. Emergence of pupae was checked at 12-hr intervals, and newly eclosed pupae were transferred to litter in a Petri dish placed in the clay vessel. Adult emergence was checked at 6-hr intervals. Duration of pupation and survival rates were determined. Dead pupae were replaced with field-collected new adults. All newly-eclosed adults emerging within a 3 day period were pooled into a single emergence class as detailed above. Five emergence classes were maintained: adults emerged during 27^th^–29^th^ day after hatching was taken as emergence class I, 30^th^–32^nd^ day period as emergence class II, 33^rd^–35^th^ day period as emergence class III, 36^th^–38^th^ day period as emergence class IV and 39^th^–41^th^ day as emergence class V.

A small clay shelter pot (7 × 20 cm) was laid on its side to prevent water entry, and a thin layer of hay was provided as shelter in each setup. Following the entry of *L. tristis* into the shelter pots, the mouths of the pots were closed with mesh net and kept closer to natural aggregations of beetles in dormitory buildings. When beetles inside the shelter pots displayed signs of arousal from dormancy by nocturnal movements and attraction to light, the mesh net was removed. The shelter pots were returned to clay vessels in the field and half filled with fresh litter to simulate movement out of the buildings. Beetles that returned to litter in clay vessels were marked with paint as detailed above. Activities of the beetles after dormancy were observed. All newly emerged larvae that developed inside the setups were removed. Activities of the old generation were observed until 100% mortality was reached. Egg hatchability, duration and mortality of larval, pupal and adult stages were recorded. Longevity was estimated as the sum of means of larval, pupal and adult durations.

**Figure 3.**
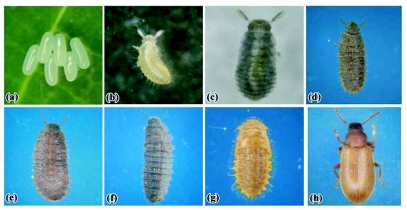
Early developmental stages of *Luprops* (a) egg mass (b) 1^st^ instar larva (c) 2^nd^ instar (d) 3^rd^ instar (e) 4^th^ instar (f) 5^th^ instar (g) pupa and (h) teneral adult.

Both laboratory and field culture of eggs were started on the same day to ensure uniformity in both setups. Neonate larvae, intermediate larval stages and pupae that died in the course of the experiment were replaced with individuals from the parallel stock culture. During the entire experimental period, fallen green leaves collected from the plantation were made available for the various stages. Exhausted leaf bits and faecal pellets were removed by upturning the vials topped with mesh net to prevent fungal contamination. Two dry folded leaves were provided in each vial as a resting and hiding substrate. A circle of filter paper moistened with drops of distilled water was placed in the petri dishes and vials used for culturing larvae and pupae.

### Behaviour during aggregation and dormancy

Activities of the aggregated beetles within the dormitory buildings were observed at 6-hr intervals until their entry into dormancy and subsequent arousal. Attraction towards water, food materials and light and qualitative assessment of reserve food materials were tested. Two hundred beetles were collected from the fresh aggregations settled in dormitory buildings and shifted to a clay vessel (7 × 30 cm) half-filled with hay and closed with mesh net. Activities during active dormancy were observed on a weekly basis. Response of beetles towards wilted leaves and moist wet filter paper placed on the mesh net, and response towards light was observed by placing a fluorescent light close to the setups from dusk to dawn. Ten randomly selected dormant beetles were dissected and the food reserves available was qualitatively assessed by examining the fat body. All tests were repeated biweekly until after arousal from dormancy.

Analysis of the flight ability of dormant beetles by throwing individuals into the air (Wolda and Denlinger 1984) failed to evoke any response. As an alternate method, 20 randomly collected beetles from the aggregations were placed on an earthen tile half covered with water in a plastic tray. Beetles were provided with a choice of remaining on the wet tile or to fly off. Tests were repeated at monthly intervals until after arousal from dormancy.

### Data analysis

Differences in egg period, larval, pupal, pre-dormancy, dormancy and post-dormancy duration and mortality during dormancy, between setups were analysed by t-test. Emergence class wise variations in mortality within and between the setups; variations in egg hatchability, larval and pupal mortality, variations in mortality during dormancy and post dormancy between the setups were tested using the χ^2^ test (Zar 2003). Gretl open source software for Windows was used in statistical analysis.

**Figure 4.**
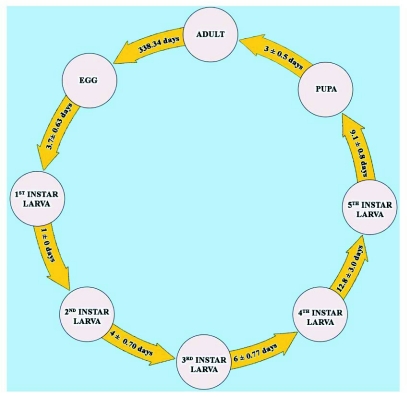
Life cycle and duration of developmental stages in laboratory setup.

## Results and Discussion

### A. Life history

The life cycle of *L. tristis* is univoltine and involves larval (five instars), pupal and adult stages following oviposition (Figures 3, 4).

**Table 1.**
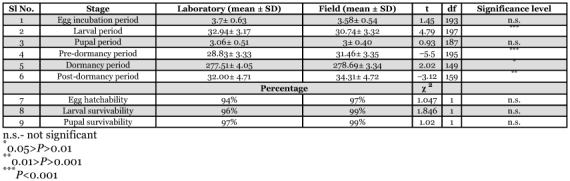
Duration of developmental stages, egg hatchability, larval and pupal mortality of *L. tristisin* laboratory and field setups.

### Oviposition and egg stages

After a preoviposition period of 5 ± 2 days (n = 30), eggs were laid singly and in batches of 2–8 on the floor of culture vials, the lower surface of dry leaves and on the mesh net topping of vials in laboratory setups. In the field, eggs were present in middle litter layers of the plantation litter, possibly to protect eggs from desiccation because of their high surface to volume ratio and inability to replace lost water during the dry season (Denlinger 1986). Eggs were laid daily or at intervals of 2–3 days. Oviposition was generally a nocturnal event except for a few incidences (n = 6) during the daytime in laboratory setups. Eggs are elongate, oval, creamy white, sticky and very delicate, measuring 1 plusmn; 0.06 mm (n = 100) in length with a distinct concave anterior pole and a convex posterior pole. The average fecundity of female was 30.6 ± 13.92 eggs (n = 30). The lowest recorded fecundity was 12 and the highest was 60. The average oviposition period was 6.1 ± 2.41 days (n=30).

**Figure 5.**
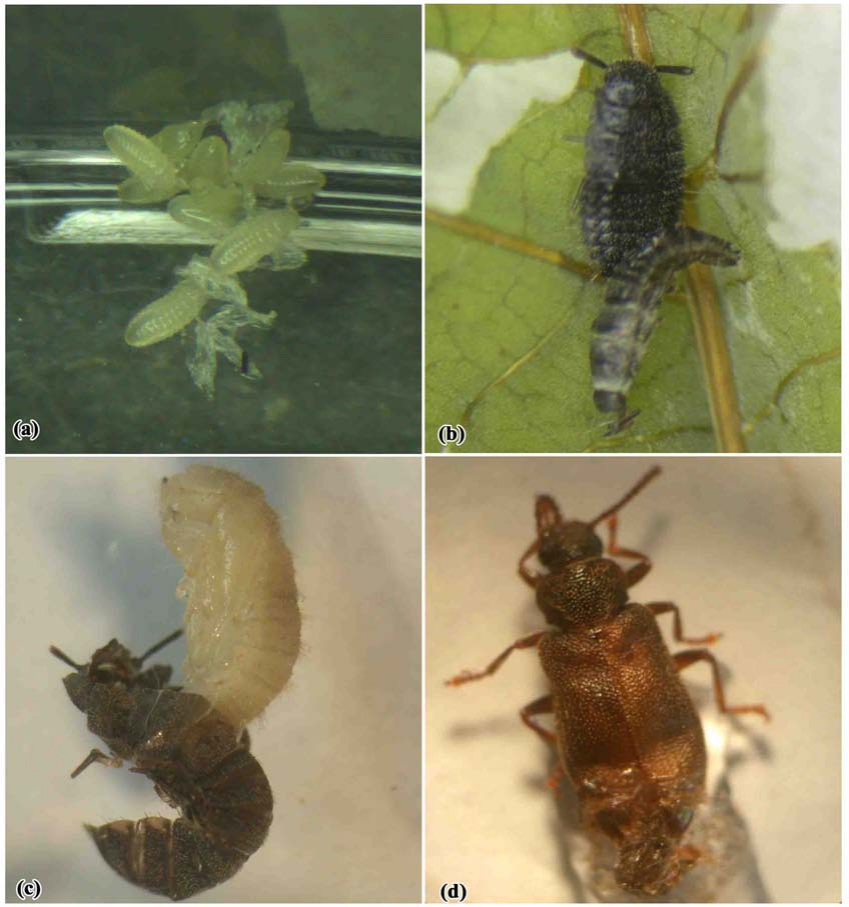
Egg hatching (a), eclosion of larva (b), pupa (c), and teneral adult (d).

The egg stage lasted 3.7 ± 0.63 and 3.5 ± 0.54 days (n = 100) in the in laboratory and field setups, respectively (Figure 4, Table 1). An estimated 97% of eggs hatched in field setups and 94% in laboratory setups, with no variation in egg hatchability (χ^2^ = 1.047, df = 1, P= 0.31) and duration of incubation (t = 1.45, df = 193, P = 0.15) between setups. No egg predation was observed in the natural setups, which we attribute to the low abundance of predatory litter arthropods in the litter floor of plantations in the early phase of litter fall (Jobi 2006).

### Larval stages

*L. tristis* has five larval instars (Table 2). In general, larvae are swift moving, flat, elongate oval, moderately rigid and dark except the first instar, which is delicate and has body color similar to that of the eggs. All newly eclosed larval instars were creamy white for a period of 1–2 hours. During molting of successive instars, which lasted for 26 ± 6 minutes (n = 100), the skin splits along a median line, the larva emerges head first, followed by thorax and legs, and the abdomen emerges last (Figure 5). Larvae were freed from exuviae within 5–15 minutes and no instances of larval inability to remove exuviae were observed. After a short period of inactivity (2–15 minutes) all stages fed preferably on wilted leaves and aggregated around water drops in both setups. The larval stage lasted was 32.94 ± 3.17 days in the laboratory and 30.74 ± 3.32 (n = 100) days in field setups. There was significant variation in larval duration in laboratory and field setups (t = 4.79, df = 197, P<0.01), which might be related to the more favorable conditions in field setups. The 1^st^ instar larval period was one day with no variation indicating that its duration was always less than 24 hours. High variability in the duration of 4^th^ instar larvae in laboratory conditions (8–21 days) in comparison to other larval instars was present, indicating that 4 instar larvae was the most sensitive stage to rear under laboratory conditions. When touched or when the resting litter particles were disturbed, all larval stages exhibited death feigning behaviour (catalepsy) lasting 1–3 minutes. Larvae were surface active from dusk to dawn and diurnally inactive beneath the cover of lower litter layers.

**Table 2.**
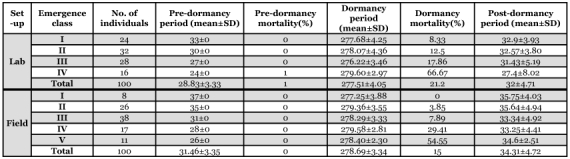
Duration of pre-dormancy, dormancy and post-dormancy and emergence class wise mortality of *L. tristis* in laboratory and field setups.

The general ignorance by the people in the region about the larval stages and their feeding activities, we attribute to the cataleptic behavior of the beetle, the pale dark color that makes them inconspicuous in litter floor and their diurnal habit of staying below dry leaves. Their nocturnal surface activities in the litter floor are in total contrast to their passive diurnal phase in the lower litter layers. Though the first instar larvae could be easily differentiated based on its color, size and short duration, differentiating 2^nd^ to the 4 larval instars in the absence of visible morphological features is impossible. Following the development of cultured individuals until pupation is the only reliable method. The survival rate of larvae (1^st^ instar to pupation in vials) was 96% in the laboratory and 99% (n = 100) under field conditions with no significant variation (χ^2^ = 1.85, df = 1, P = 0.17).

### Pupal stage

**Figure 6.**
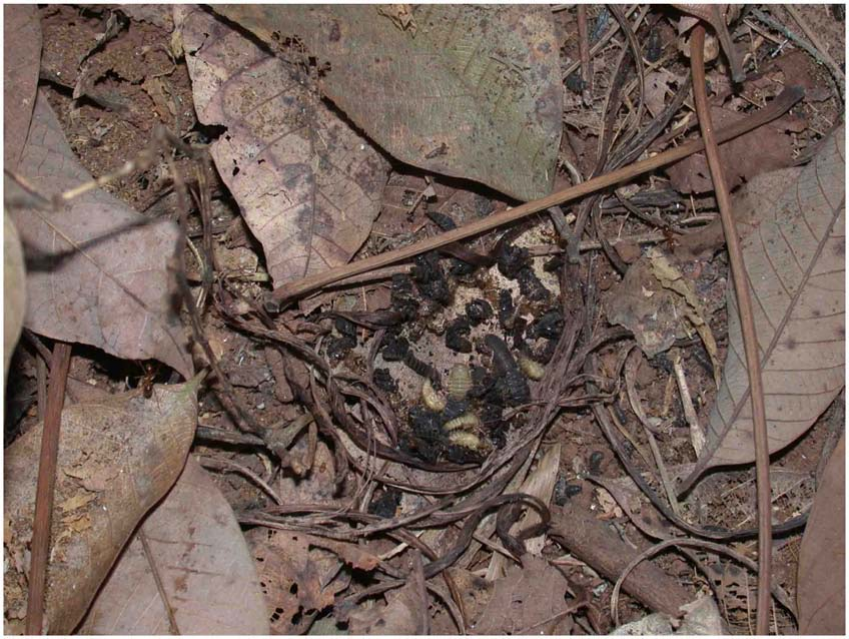
Cluster of V^th^ instar larvae in a quiescent state prior to pupation and newly emerged pupae in the litter floor of rubber plantation.

Pupae are naked and are not of the exarate or obtect type (Evans 1975). Prior to pupation 5^th^ instar larvae aggregated in groups of 2–10 in the field setup and in groups of 2–20 under natural conditions, in a pre-pupal quiescent phase for 4–5 hrs. The duration of the pupal stage was 3.06 ± 0.51 days and 3 ± 0.40 days (n = 100) in laboratory and field setups respectively (Table 1). Survival probability of pupa was 97% (n = 100) in laboratory setups and 99% (n = 100) in field setups without significant variation in pupal duration (t = 0.93, df = 187, P = 0.36) and survival probabilities (χ^2^ = 1.020, df = 1, P = 0.31) in both setups. Clusters of 2–20 creamy white pupae (Figure 6) are present in middle litter layers in field, which is likely to provide protection from desiccation. In laboratory setups, they hid behind leaf particles and pupated singly. Pupal duration did not vary between the isolated laboratory conditions and the crowded field conditions. These observations are contrary to the reported inhibition of pupation in crowded conditions in other tenebrionids (Tschinkel and Wilson 1971).

### Adult stage

The adult stage of *L. tristis* involved 3 distinct phases, a pre-dormancy and post-dormancy period both in litter habitat and an intermediate dormancy period in shelters. Adults took refuge below litter diurnally and were surface active nocturnally.

#### 1. Pre-dormancy phase:

Newly eclosed yellowish brown (teneral) adults were inactive and did not feed until they attained a dark colour 3–4 hours of after eclosion. Subsequently, they fed preferentially on wilted tender leaves. In the absence of visible sexual dimorphic characters and overlapping male and female size ranges (6.7–8.0 mm and 7.0–8.5 mm in male and female respectively, n=30) makes the dimorphism present on the 8th sternal notch (Vinod et al. in press) the sole mechanism to differentiate sexes. The pre-dormancy period lasted for 28.83 ± 3.33 days in laboratory setups (n = 99) and 31.46 ± 3.35 (n = 100) days in field setups with significant variation (t = -5.50, df = 195, P<0.01) (Table 1). Mortality during the phase was low in both setups (no mortality in field setups and 1% mortality in laboratory setups). Based on duration of the pre-dormancy phase, adults in field setups were categorized under 5 emergence classes and under 4 emergence classes in laboratory setups (Table 2). 64% of the adults in the laboratory and 60% of adults in field setups belonged to emergence class II and III. Intrapopulation variation in prediapause period arises from the variation in larval duration and leads to differences in the active feeding time available prior to dormancy. Emergence class I in laboratory setups had an average of 9 days more feeding time in comparison to class IV, and class I in the field setup had an average of 11 days more feeding time than class V (Table 2). Abdominal cavities of *L. tristis* in emergence class I and II were more fully loaded with fat body reserves during the last phase of dormancy than in class IV and V.

#### 2. Dormancy phase:

*L. tristis* in field setups entered the shelters (small wooden boxes and pots) in response to the first short duration summer rain itself (April 4, 2005) and those in laboratory setups in response to simulated wet conditions. Within the shelters, the beetles were nocturnally surface active for 8–11 days in field and laboratory placed setups, with a strong attraction towards light and fresh leaf materials, as observed by their aggregation on nets and attempts to crawl out through the mesh sieves in laboratory setups. Subsequently they clustered and entered into a prolonged inactive state for 9 months (Table 1). The first indication that dormancy is coming to an end is nocturnal dispersal within the shelters, which is initiated by a few individuals and is followed by the rest within 2–3 days. Nocturnal dispersal lasted for 15–17 days (December 29^th^ 2005- January 15^th^ 2006). Diurnally, they remained inactive in the corners of the shelter in small groups. No beetles from the shelters returned to the litter at this stage.

This prolonged period of inactivity and aggregation is categorized as the active state of dormancy. The periods of short duration nocturnal dispersal and diurnal passivity prior to and after dormancy is characterized as the refractory and termination periods respectively (Mansingh 1971; Danks 1987). During the refractory period, beetles were attracted to food materials while they were not during the active dormancy phase. Dormancy lasted for 277.51 ± 4.05 days in laboratory setups and 278.69 ± 3.34 days in field setups with significant variation between the setups (t = 2.02, df = 149, P = 0.04). The refractory period lasted for 8 to 10 days in the laboratory and 8 to 11 days in the field and the termination phase lasted for 6 to 15 days in the laboratory and 6 to 17 days in the field. Mortality during dormancy was 15% in the field and 21.2% in laboratory setups with no significant variation (χ^2^ = 1.71, df = 1 , P = 0.19). Emergence class variation in mortality was significant (χ^2^ = 22.57 and 21.72, P< 0.01 in the laboratory and field respectively) with low mortality in long duration emergence class (classes I–III; 8.3% and 0 respectively in laboratory and field setups) and higher mortality in short duration emergence classes (class IV in both setups and V in field setups; 66.7% and 54.5% respectively in laboratory and field setups) in both the experiment setups (Table 2). Those that attained adulthood early had a longer feeding duration and, hence, may have had a higher fitness than adults that emerged later. Generally, mortality during dormancy is very low in natural populations (Wolda and Denlinger 1984; Danks 1987). High mortality in the short emergence classes in experimental setups and the high number of dead beetles on the floor of wooden ceilings in residential buildings indicates high mortality during dormancy in *L. tristis.* At no stage during the analysis did we observe any insectivores such as wall lizards (*Hemidactylus* sp.), spiders or ants especially the aggressive *Oecophylla smaragdina* (Fabricius), feeding either on adults nor larvae. Hence, we attribute the high mortality in natural setups to the presence of higher number of beetles in shorter emergence class (shorter pre-dormancy period) with low feeding duration and not to predation.

The long duration dormancy shown by *L. tristis* involving adults of various emergence classes does not fall under either quiescence or diapause. The term quiescence is used in situations with a shorter period and no induction phase and with active feeding throughout the stage (Mansingh 1971; Danks 1987). *L. tristis* does not diapause either, as diapause is preceded by a long preparatory phase during which energy reserves are accumulated at a specific stage in life cycle. Entry of beetles with both short and long durations of pre-dormancy into active dormancy, irrespective of the active feeding time available indicates that preparations for diapause by accumulation of energy reserves prior to active dormancy is not mandatory in *L. tristis*. In classical dormancy, the preparatory phase is succeeded by a typically long refractive phase and the insects cannot terminate diapause and continue morphological development even if exposed to optimal environmental conditions during this stage (Mansingh 1971; Tauber and Tauber 1976; Denlinger 1986, 2002; Danks 1987). However, in the present case, beetles were readily attracted to food resources and water during the short refractory phase. Adults of all emergence classes irrespective of the active feeding duration available entered into dormancy without a preparatory phase nor an induction period in response to an unexpected environmental token stimulus, the short mid summer rainfall that mediated wetness in the rubber plantation litter floor. During the short refractory period, beetles were readily attracted to tender leaves and dry litter conditions. These observations weighed in favor of our categorization of dormancy in *L. tristis* as oligopause and not as diapause, which is intermediate between quiescence and diapause (Mansingh 1971; Danks 1987; Leather et al. 1995).

Recognizing the environmental stimuli leading to and arousal from dormancy is a difficult task for many tropical insects (Denlinger 1986). Moisture, photoperiod, temperature, food availability and food quality (Mansingh 1971; Tauber and Tauber 1976; Masaki 1980; Hunter and McNeil 1997; Tanaka 2000; Danks 2003; Sato 2003; Nahrung and Allen 2004; Wang et al. 2006; Nakamura and Numata 2006) are the recognized dormancy induction cues. Sudden annual mass entry of huge swarms of *L. tristis* into residential buildings following the first summer rains is a widely recognized and familiar event in the rubber plantations belts of the region. The absence of beetles in the litter floor of plantations following the first summer rains and the entry of beetles into dry litter conditions in the experiment setups, together with the general affinity of tenebrionids toward dry conditions (Watt 1992; Endrody-Younga and Tschinkel 1993) indicates that wetness in litter habitat leads to the departure of beetles and migration towards shelters. Additionally, no beetles in stock cultures maintained in laboratory setups and away from rainfall-mediated wetness enter into dormancy (unpublished data) even after the onset of monsoon rains later in the month of June. Our current data strongly suggest that rainfall mediated wetness from summer showers is the oligopause inducing cues for *L. tristis*. However further empirical studies considering the above stated environmental factors recognised as the cues for diapause in insects are necessary.

**Table 3.**
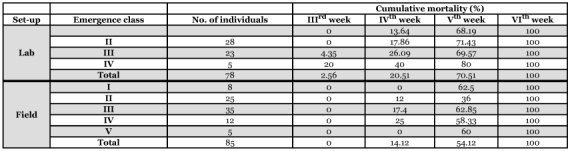
Emergence class wise mortality of *L. tristis* during post dormancy phase in laboratory and natural set ups.

Synchrony of diapause arousal and subsequent field return of beetles with the beginning of annual leaf fall in the rubber plantations in the region points towards the following probabilities. Deciduous trees shed leaves well in advance of the onset of the dry season as a mechanism to prevent water loss (Santiago et al. 2004, Santiago and Mulkey 2005, Sundarapandian et al. 2005). Subsequent dry environmental conditions in the region during this phase, along with substantial leaf fall provide a suitable habitat and food source for the beetles. We suggest that the return of dry conditions following the prolonged monsoon season, and the related variation in moisture, photoperiod and temperature in the region are the environmental cues leading to the arousal of *L. tristis* from oligopause. The seasonal coincidence of a thick layer of freshly fallen litter in rubber plantations both as a food resource and as a habitat facilitates the return of post-dormant beetles to the field.

#### 3.Post-dormancy phase:

Following the termination phase, adults returned to the litter in laboratory setups in batches during the first and second weeks of January (December 29^th^ —January 15^th^), which lasted for 6 to 17 days. The size of aggregations in residential buildings decreased from the week of December 4^th^ onwards and no beetles were present in the buildings after the 3^rd^ week of January (January 20). Beetles were less common on the litter floor during the first week of December and their number steadily increased in the following weeks and peaked in the 4^th^ week of January (200 ± 25.6/m^2^; n = 30) (unpublished data). Post-dormancy beetles that returned to the litter habitat were attracted to water drops on leaves (dew drops in plantations) and fed on the outer edges of dry leaves until tender leaves were available that emerged synchronous with the premature fall of tender leaves in plantations. Following an 18–25 day period of intensive feeding in laboratory setups, mating pairs appeared with the male climbing on the back of the female and pressing the abdomen down on the abdomen of the female to engage his genitalia with hers. Neither mating pairs nor egg production by the newly emerged adults were recorded either in the laboratory setups or from natural conditions prior to or during dormancy. Total absence of reproductive activities during pre-diapause and diapause periods, and the initiation of mating activities after a brief period of intense feeding following post diapause arousal, suggest the following probabilities. Either the reproductive organs in *L. tristis* do not mature until the field return or the beetles are undergoing reproductive diapause (Danks 1987; Mansingh 1971), similar to what is reported during the dormancy in *S. rotundus* (Wolda and Denlinger 1984).

Post-dormancy duration was 34.31 ± 4.72 days (n = 85) in laboratory and 32 ± 4.71 days (n = 78) in field setups with significant variation in the post-dormancy period (t = -3.12, df = 159, P = 0.002) (Table 1). No mortality was recorded in the first two weeks of post-dormancy phase in either experiment setup, and up to the third week in field setups. Subsequently, significantly high mortality was recorded during the 5 week in laboratory setups (χ^2^ = 4.64, df = 1, P = 0.03) and 100% mortality in both setups (Table 3). The adult stage duration was 338.34 days with a pre-dormancy period of 28.83 ± 3.33 days, a post-dormancy period of 32.00 ± 4.71 days and an intermediate dormancy period of 277.51±4.05days in laboratory setups. In field setups, the adult stage duration was 344.46 days with a pre-dormancy period of 31.46 ± 3.35 days, a post-dormancy period of 34.31 ± 4.72 days and an intermediate dormancy period of 278.69 ± 3.34 days. Longevity of *L. tristis* was 374.34 days in laboratory setups and 378.2 days in the field setups.

**Figure 7.**
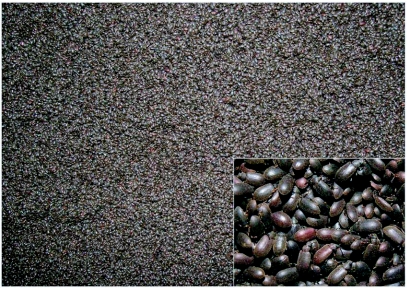
Aggregated dormant beetles settled on the wall and gaps of a residential building.

We attribute the comparatively higher mortality and shorter post dormancy duration in laboratory setups to the more favourable conditions available in natural setups during the dry summer conditions and to the shorter larval phase and the additional 2.63 days of active feeding time available to the adults in field setups during the prediapause phase. Future studies involving laboratory culture of *Luprops* should make note of the effect of the duration of larval and pre-dormancy phase on mortality, longevity and fitness in subsequent phases.

### B. Features of aggregations

**Figure 8.**
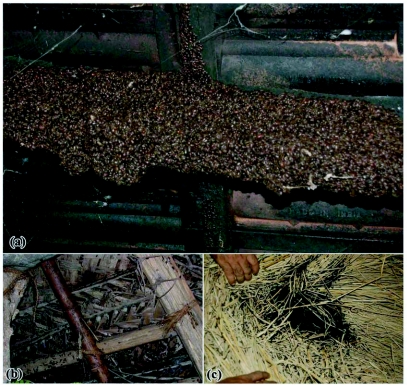
Aggregated dormant beetles settled on (a) wooden frames of tile roofed buildings (b) thatched sheds made of coconut fronds and (c) hay stack in cattle shed.

Huge aggregations of *L. tristis* were nocturnally active and were attracted to light for up to 20 days after entering the buildings from plantations following first summer rains (Figure 7). They hid behind crevices and wall hangings in living rooms during the day. Subsequently they moved behind the dark corners in wooden ceilings and attics and entered into dormancy. Aggregating beetles were 3–4 layers deep and occasionally honey comb-like aggregations hung precariously from the wooden supports (Figure 8). Our attempts to separate the members present in outer layers showed that the beetles clung very strongly to one another in the aggregation. Dormant beetles showed no response towards light nor towards leaf materials. Except for a short duration (2–3 hours) massive diurnal dispersal within the buildings on one rainy afternoon following a 5 day rainless, hot, and humid period (November 1^st^ 2005), dormant beetles were observed to be immobile. The beetles did not lose their capacity for flight. Their flight muscles did not degenerate, as was evidenced by the mass flight of beetles in response to experimental wetting of the tiles. This is contrary to reports in *S. rotundus* (Wolda and Denlinger 1984). How and why they maintain flight ability and flight muscles during dormancy, which is costly, and energy consuming remains unanswered? We suggest that, unlike in diapause, degeneration of flight muscles and loss of flight capacity may not be taking place during oligopause.

Aggregation during diapause is common among insects as an energy and water conserving mechanism (Wolda and Denlinger 1984; Denlinger 1986; Danks 1987; Leather et al. 1995). Though water was available during the dormancy period, beetles never left the aggregations and shelters in search of drinking water. So, aggregation in *L. tristis* is more likely to be a mechanism to insulate them from the cold wet conditions prevailing in the region during the prolonged monsoon rains. During the last phase of dormancy, the fat reserves were totally absent in abdomen. As no other food resources were available to the beetles in the experimental setups, they rely exclusively on the fat reserves in their abdomen during dormancy.

### Conclusions

We present details of the biology and dormancy of *L. tristis* as an example of a species integrating dormancy and aggregations in a tropical region. Oligopause in response to early summer showers provides the following advantages to *L. tristis*, which are recognized as the general advantages of dormancy (Danks 2002, 2006). It leads to protection from adverse habitat conditions, synchronized emergence of physically fit adult reproductive population in a subsequent season, optimization of the time of reproduction, synchronization of the feeding stages (larval and adults) with food resources (litter in the fall) in rubber plantations, prevention of the arrival of vulnerable early developmental stages during the monsoon periods, and synchronization of the feeding stages and reproductive stages. Our results reveal that the following factors have contributed to the enormous success of *L. tristis* in the region *viz*., 1) capacity of oligopause during the seasonally inimical wet seasons, 2) arousal from dormancy synchronized with the return of dry seasons, 3) annual litter fall in the rubber plantations, 4) completion of sensitive stages in the life history before return of inimical conditions and 5) the amazingly high survivability of all early developmental stages *viz*., larvae and exposed pupae.

We expect that the results will lead to further research to answer the following questions about *L. tristis*.

1. Since generations are not overlapping and the same buildings are regularly infested leaving neighboring buildings uninfested, raises the question of how do the newly emerged adults with no previous experience select the same buildings used by the previous generation for aggregation?
2. What attracts them to aggregate in specific buildings and thatched sheds successively for years even after application of both organic and inorganic repellents and insecticides?
3. Are they univoltine with longer dormancy or have a facultative univoltinism necessitated by the absence of dry litter habitat conditions and depleting food resources?
4. When do the gonads become mature and functional? Is it a case of reproductive diapause or delayed maturity?
5. How do the variations in emergence -class structure of the individuals affect the fecundity and fitness of post-diapause beetles?
6. What is the reason for high mortality during dormancy in the short-duration emergence classes in experimental setups and in natural aggregations? What is the optimum and minimum duration of feeding time required before dormancy?
7. Does the maintenance of flight muscles by such a large aggregations of beetles throughout the period of dormancy indicate that the accumulated fat reserves are of sufficiently high nutritive quality to provide energy for prolonged periods?
